# Brazilian *Bothrops* and *Crotalus* Snake Venoms as a Source of Antiprotozoal Agents: Unlocking Toxins for Therapeutic Development

**DOI:** 10.3390/toxins18090405

**Published:** 2026-09-21

**Authors:** Tulio Custódio Reis, Ana Clara Lunardi Yagi, Eloise T. Filardi, Marcela Romanazzi, Michele Ferreira da Silva Mela, Rui S. Ferreira Júnior, Felipe A. Cerni, Márcia A. S. Graminha, Manuela B. Pucca

**Affiliations:** 1 Graduate Program in Bioscience and Biotechnology Applied to Pharmacy, School of Pharmaceutical Sciences, São Paulo State University (UNESP), Araraquara 19060-900, São Paulo, Brazil; tulio.reis@unesp.br (T.C.R.); ana.cl.yagi@unesp.br (A.C.L.Y.); e.filardi@unesp.br (E.T.F.); marcela.romanazzi@unesp.br (M.R.); michele.mela@unesp.br (M.F.d.S.M.); 2 Center for the Study of Venoms and Venomous Animals (CEVAP), São Paulo State University (UNESP), Botucatu 18619-002, São Paulo, Brazil; rui.seabra@unesp.br; 3 Medical School, Federal University of Roraima (UFRR), Boa Vista 69310-000, Roraima, Brazil; felipe_cerni@hotmail.com; 4 Department of Clinical Analysis, School of Pharmaceutical Sciences, São Paulo State University (UNESP), Araraquara 19060-900, São Paulo, Brazil

**Keywords:** antileishmanial agents, Brazilian snakes, neglected tropical diseases, venoms, *Bothrops*, *Crotalus*

## Abstract

Leishmaniasis and Chagas disease are neglected tropical diseases that continue to pose significant public health challenges, highlighting the need for new therapeutic strategies. Brazilian snake venoms represent a valuable and underexplored source of bioactive molecules with diverse pharmacological activities and promising therapeutic potential. In this context, the present study aimed to investigate the in vitro antiprotozoal potential of venoms from *Bothrops* and *Crotalus* species, including *Bothrops jararacussu*, *Bothrops pauloensis*, *Bothrops alternatus*, *Bothrops leucurus*, *Bothrops atrox*, *Bothrops jararaca*, *Crotalus durissus terrificus*, and *Crotalus durissus ruruima*, metalloprotease (MTL)- and crotoxin (CTX)-enriched, against *Leishmania* spp. and *Trypanosoma cruzi*. Crude venom protein profiles were analyzed by SDS-PAGE, and cytotoxicity was assessed in differentiated C2C12 myotubes and murine peritoneal macrophages. Antiprotozoal activity was evaluated against extracellular parasite forms, including promastigotes of *Leishmania amazonensis* and *Leishmania infantum* and epimastigotes of *T. cruzi*. Venoms exhibiting the highest selectivity indices (SIs), calculated using murine macrophage cytotoxicity data, were further tested against intracellular amastigotes of *L. amazonensis*. Among the tested samples, *B. jararacussu* venom showed activity against promastigotes of both *L. amazonensis* (IC_50_ = 2.9 µg mL^−1^) and *L. infantum* (IC_50_ = 2.8 µg mL^−1^), as well as in epimastigotes of *T. cruzi* (IC_50_ = 15.5 µg mL^−1^). The MTLand CTX pools from *C. d. ruruima* venom exhibited the highest activity against promastigotes of *L. amazonensis*, with IC_50_ values of 1.6 and 3.8 µg mL^−1^, respectively. Against intracellular amastigotes, MTL and CTX showed IC_50_ values of 4.1 and 2.5 µg mL^−1^, respectively, with selectivity indices (SIs) of 73.1 and 120, respectively. Overall, these findings provide preliminary evidence for the antiparasitic potential of snake venom-derived molecules, supporting further characterization and in vivo evaluation.

## 1. Introduction

Brazil is recognized as the most biodiverse country in the world and ranks first among the world’s megadiverse countries. It harbors approximately 15–20% of global biological diversity, representing an exceptional reservoir of biological resources for the discovery and development of novel bioactive molecules [1,2]. The country encompasses six major terrestrial biomes, Amazon rainforest, Cerrado, Atlantic Forest, Caatinga, Pantanal, and Pampa, each characterized by distinct ecological conditions and exceptional species richness, including high levels of endemism [1]. This remarkable biological diversity extends to the fauna of venomous animals, which constitutes one of the richest assemblages in the world, encompassing numerous species of snakes, scorpions, spiders, and other arthropods of medical relevance [3]. Envenomation by these animals represents a substantial public health burden in Brazil. Between 2007 and 2019 alone, more than 1.8 million cases of envenomation and 3506 associated deaths caused by terrestrial venomous animals were reported to the Brazilian Notifiable Diseases Information System (SINAN) [4,5].

Among venomous animals of medical importance in Brazil, snakes occupy a prominent position. Approximately 62 species of venomous snakes are recognized in the country, classified into four groups of clinical relevance: Bothropic (genera *Bothrops* and *Bothrocophias*), Crotalid (genus *Crotalus*), Lachetic (genus *Lachesis*), and Elapidic (genus *Micrurus*) [6]. Snakebites account for approximately 29,000 cases and 125 deaths annually in Brazil, with the Bothropic group responsible for approximately 85 to 86% of all accidents [7].

Within this group, species such as *Bothrops jararacussu*, *B. pauloensis*, *B. alternatus*, *B. leucurus*, *B. atrox*, and *B. jararaca* are widely distributed across Brazilian biomes and display considerable interspecific variation in venom composition, toxicity, and pharmacological activities [7]. The Crotalid group (e.g., rattlesnakes), represented in Brazil by six subspecies of *Crotalus durissus*, is responsible for approximately 9% of snakebite accidents and is associated with severe systemic manifestations, including neurotoxicity and myotoxicity, primarily attributed to crotoxin, the major toxic component of these venoms, and crotamine. Among the Crotalic subspecies, *C. d. terrificus* is the most widespread in Brazil, while *C. d. ruruima* is restricted to the northernmost state of Roraima and is associated with some of the highest lethality rates among rattlesnake envenomings in the country [8].

Snake venoms are complex biochemical cocktails comprising proteins and polypeptides that account for approximately 95% of their dry weight, organized into several major protein families, including phospholipases A2 (PLA2), snake venom metalloproteases (SVMPs), L-amino acid oxidases (LAAOs), C-type lectins, serine proteases (SVSPs), and low-molecular-weight peptides, each exhibiting distinct and often overlapping pharmacological activities [9]. The breadth of biological activities harbored by these molecules, spanning neurotoxic, myotoxic, hemotoxic, antimicrobial, antitumoral and antiparasitic effects, positioned snake venoms as a valuable reservoir for the discovery of novel pharmacological scaffolds [10]. In recent decades, several clinically approved drugs have been derived from or inspired by venom components, such as captopril (from *B. jararaca* bradykinin-potentiating peptides), eptifibatide (from the pygmy rattlesnake disintegrin barbourin), and tirofiban (based on the echistatin peptide from *Echis carinatus* venom), underscoring the translational relevance of this research field [11]. More recently, snake venoms have been increasingly investigated for their antiprotozoal properties, emerging as a promising strategy to address the pressing need for novel therapeutic agents against neglected tropical diseases, including leishmaniasis and Chagas disease [12].

Leishmaniasis is a group of vector-borne diseases caused by obligate intracellular protozoan parasites of the genus *Leishmania*, transmitted to vertebrate hosts through the bites of infected female phlebotomine sandflies (Diptera: Psychodidae) [13]. Classified by the World Health Organization (WHO) as a neglected tropical disease (NTD), leishmaniasis affects populations in over 90 endemic countries, with an estimated 700,000 to 1 million new cases reported annually and visceral leishmaniasis alone responsible for 20,000 to 30,000 deaths per year [14]. The disease presents three main clinical forms, cutaneous (CL), mucocutaneous (MCL), and visceral leishmaniasis (VL), each determined by the infecting *Leishmania* species, the host immune response, and the geographic context of transmission [15]. VL, the most severe and potentially fatal form if left untreated, is caused primarily by *Leishmania infantum* in the Americas and *Leishmania donovani* in East Africa and South Asia, with Brazil, India, and East Africa collectively accounting for the majority of global VL cases [16]. In Brazil, *L. infantum* is the exclusive etiological agent of VL, transmitted mainly by the sandfly *Lutzomyia longipalpis* in urban and periurban settings, where domestic dogs (*Canis familiaris*) serve as the primary reservoir host, sustaining the transmission cycle and facilitating the spread of the parasite to human populations [17]. Despite ongoing national control programs, VL continues to expand geographically across Brazil, driven by a complex interplay of factors including poverty, inadequate sanitation, deforestation, and unplanned urbanization [16].

In parallel, American tegumentary leishmaniasis (ATL), caused in Brazil predominantly by *Leishmania* (*Viannia*) *braziliensis*, *L.* (*V.*) *guyanensis*, and *Leishmania* (*Leishmania*) *amazonensis*, represents another significant public health challenge, with approximately 30,000 new cases reported annually, manifesting across a broad clinical spectrum ranging from localized cutaneous ulcers to severe mucosal destruction and diffuse cutaneous disease, particularly in rural, forest-edge, and peri-Amazonian environments [18].

Chagas disease, caused by the protozoan *Trypanosoma cruzi*, is another major neglected tropical disease (NTD) in the Americas; first described by Carlos Chagas in 1909, it is currently endemic in 21 Latin American countries [19]. According to the World Health Organization, the estimated prevalence is 6 to 8 million *T. cruzi* infection cases, with over 100 million people at risk of infection and more than 10,000 deaths reported annually [20]. Contaminative vector-borne transmission remains the primary route of infection via the triatomine vector (Hemiptera: Reduviidae), popularly known as the “kissing bug” [21]. However, other transmission routes are known, ranging from congenital and transfusion-related transmission to ingestion of contaminated food, as well as laboratory accidents (though less frequent) [22]. The natural history of Chagas disease is characterized by an initial acute phase—typically asymptomatic or presenting with nonspecific manifestations—followed by a prolonged indeterminate chronic phase that can persist for decades without detectable parasitemia [23]. Despite advances in vector control and blood screening since the 1990s, Chagas disease remains a public health challenge due to oral transmission, migration, and the persistent population of individuals in the chronic phase [19,24].

For leishmaniasis, treatment relies on a small number of drugs, each carrying substantial limitations. Pentavalent antimonials sodium stibogluconate and meglumine antimoniate have historically been the first-line agents, but their use is increasingly compromised by widespread resistance, particularly in the Indian subcontinent, as well as by severe toxicity, the need for prolonged parenteral administration, and requirements for hospitalization and close monitoring. Amphotericin B, although highly effective, with cure rates approaching 100%, is limited by significant nephrotoxicity, infusion-related reactions, high cost, and the necessity of cold chain logistics that are frequently unavailable in resource-limited endemic settings [25]. Miltefosine, the only oral antileishmanial drug currently approved, presents challenges including teratogenicity, gastrointestinal and renal toxicity, regional variability in efficacy, and emerging resistance, while paromomycin has shown inconsistent outcomes across different geographic regions and *Leishmania* species [24].

For Chagas disease, the therapeutic landscape has remained essentially unchanged for over five decades: benznidazole and nifurtimox, both developed in the 1960s, are the only drugs currently recommended for etiological treatment under WHO authorization [26]. Collectively, the toxicity, limited efficacy in chronic stages, restricted accessibility, and the absence of a preventive vaccine for either disease underscore the urgent and unmet need for the discovery of new, safer, and more effective antiprotozoal compounds, particularly those derived from natural sources [27,28].

Notably, crude venoms and isolated fractions from Brazilian species of the genera *Bothrops* and *Crotalus* have shown significant dose-dependent inhibitory effects against promastigotes of *L. amazonensis* and *L. infantum*, epimastigotes of *T. cruzi*, and intracellular amastigotes of *L. amazonensis*, positioning these venoms as particularly relevant sources of candidate molecules for the development of new antiprotozoal drug prototypes [29].

Considering that the available evidence remains limited to a restricted number of species, venom components, and parasite developmental stages, the objective of this study was to investigate the antiprotozoal potential of eight Brazilian *Bothrops* and *Crotalus* species. We hypothesized that these venoms and their enriched fractions contain bioactive components with activity against *Leishmania* spp. and *T. cruzi*.

The present study investigated the antiprotozoal potential of venoms obtained from eight medically relevant Brazilian snake species and venom-derived pools, including *B. jararacussu*, *B. pauloensis*, *B. alternatus*, *B. leucurus*, *B. atrox*, *B. jararaca*, *C. d. terrificus*, as well as a metalloprotease-enriched pool (MTL) and a crotoxin-enriched pool (CTX) derived from and *C. d. ruruima*. These venoms were systematically evaluated against differentiated myotube cells and murine peritoneal macrophages, as well as distinct developmental stages of clinically important protozoan parasites, including promastigotes of *L. amazonensis* and *L. infantum*, epimastigotes of *Trypanosoma cruzi*, and intracellular amastigotes of *L. amazonensis*. Collectively, these findings contribute to the expanding field of toxinology-driven bioprospection and reinforce the therapeutic potential of Brazilian snake venoms as valuable sources of novel molecules for the development of antiparasitic strategies targeting neglected tropical diseases.

## 2. Results

### 2.1. Venom Profiling by Electrophoresis

**Figure 1:**
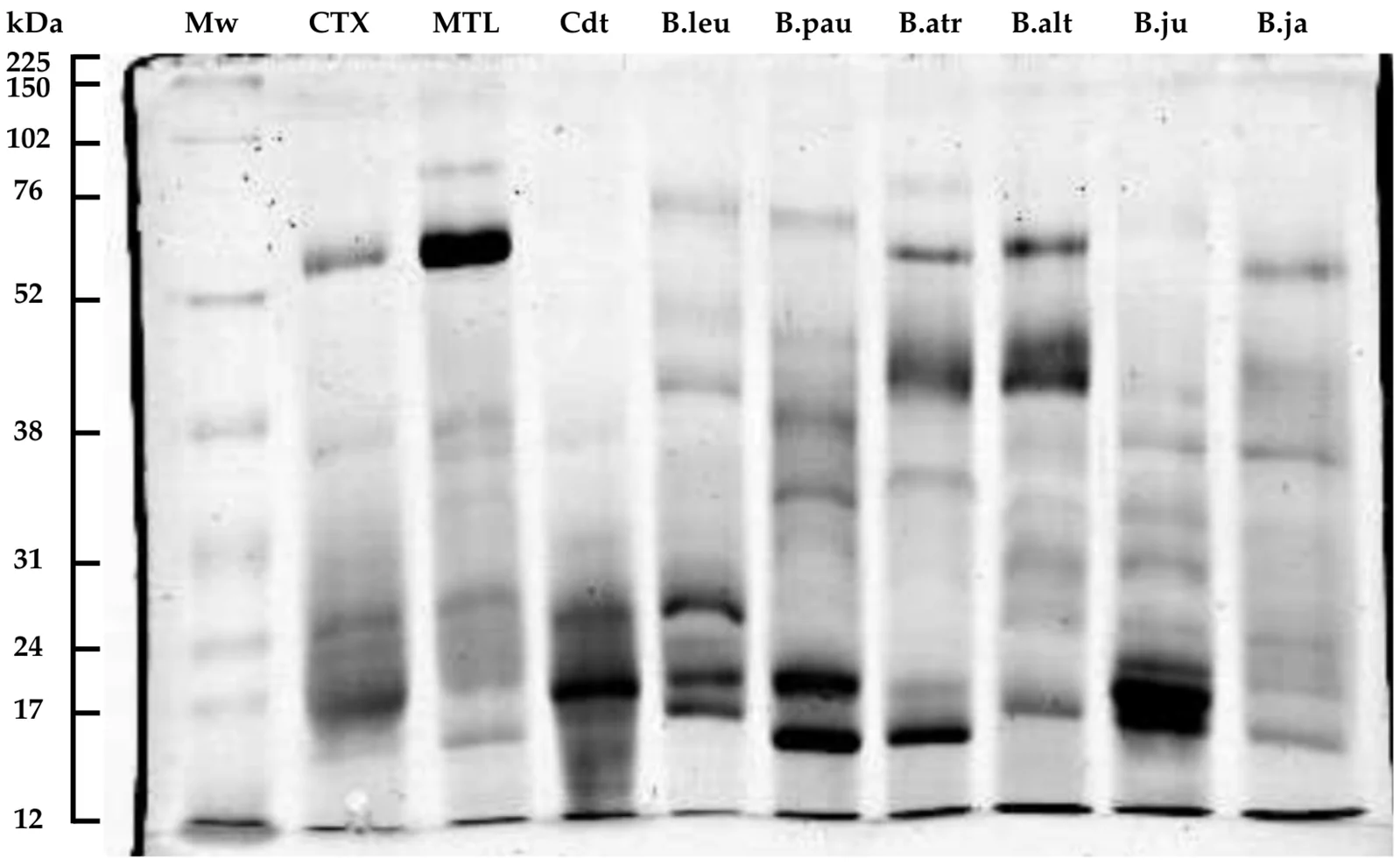
Electrophoretic profiles of snake venoms analyzed by SDS-PAGE (12%) under denaturing and reducing conditions. Sample identification: Mw, molecular weight marker; CTX, *C. d. ruruima* crotoxin-enriched pool; MTL, *C. d. ruruima* metalloprotease-enriched pool; Cdt, *C. durissus terrificus*; B.leu, *B. leucurus*; B.pau, *B. pauloensis*; B.atr, *B. atrox*; B.alt, *B. alternatus*; B.ju, *B. jararacussu*; and B.ja, *B. jararaca*. A total of 20 µg of each venom sample was loaded per lane. Coomassie Brilliant Blue R-350 staining. The Amersham ECL Rainbow Marker—Full Range (Sigma, Little Chalfont, UK) was used as the protein marker.

The protein profiles of the crude venoms were initially evaluated by sodium dodecyl sulfate–polyacrylamide gel electrophoresis (SDS-PAGE) under denaturing and reducing conditions. Protein separation was performed using a 12% resolving gel and a 5% stacking gel, with 20 µg of each crude venom loaded per lane. Electrophoresis was conducted at a constant voltage of 120 V, and protein bands were visualized by Coomassie Brilliant Blue R-350 staining (Figure 1). The resulting electrophoretic patterns were used to characterize the protein complexity of the crude venoms. These profiles also provided a qualitative basis for interpreting the observed antiprotozoal activities against *L. amazonensis*, *L. infantum*, and *T. cruzi*.

### 2.2. Cytotoxicity Against Differentiated C2C12 Myotubes

**Figure 2:**
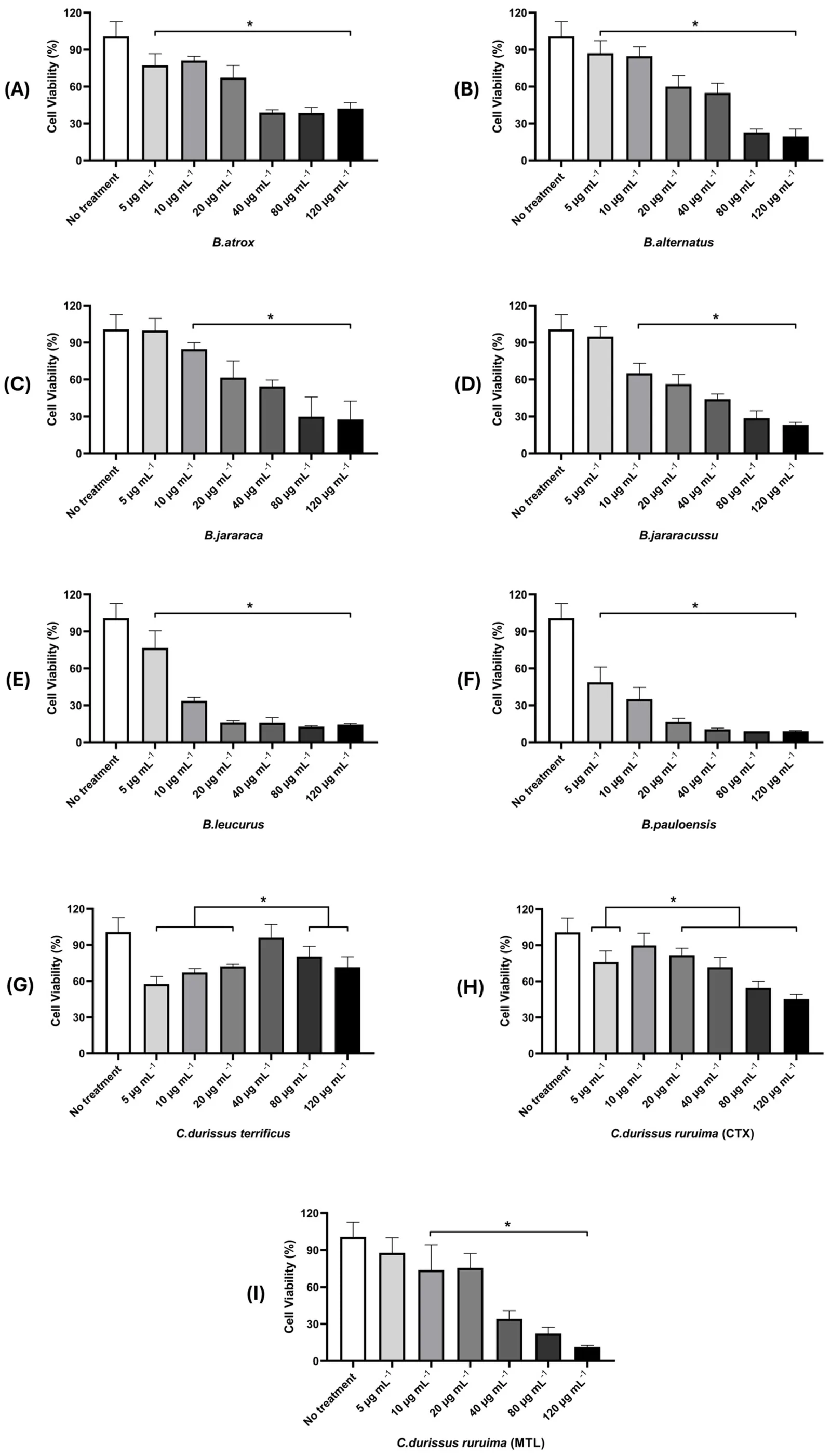
Cytotoxicity evaluated in differentiated C2C12 myotubes. In this assay, the venoms of (**A**) *B. atrox*, (**B**) *B. alternatus*, (**C**) *B. jararaca*, (**D**) *B. jararacussu*, (**E**) *B. leucurus*, (**F**) *B. pauloensis*, (**G**) *C. durissus terrificus*, (**H**) *C. durissus ruruima* (CTX), and (**I**) *C. durissus ruruima* (MTL) were evaluated at increasing concentrations ranging from 5.0 to 120 µg mL^−1^. An unpaired *t*-test was used to compare the percentage of cell viability at each concentration with its respective untreated control group. An asterisk (*) indicates a statistically significant difference compared with the untreated group (*p* < 0.05).

For this assay, the venoms of *B. atrox*, *B. alternatus*, *B. leucurus*, and *B. pauloensis* induced a significant reduction in C2C12 myotube viability at all tested concentrations (5–120 µg mL^−1^) when compared with the untreated control group. In contrast, the venoms of *B. jararaca*, *B. jararacussu*, and *C. d. ruruima* (MTL) significantly reduced cell viability only at concentrations up to 10 µg mL^−1^, whereas distinct cytotoxicity profiles were observed for the remaining venoms evaluated (Figure 2). Notably, venoms from species belonging to the genus *Bothrops* exhibited pronounced myotoxic effects, particularly at the highest concentrations tested (80 and 120 µg mL^−1^). The elevated cytotoxicity observed for these venoms is likely associated with the combined action of myotoxic phospholipases A_2_ (PLA_2_s), snake venom metalloproteinases (SVMPs), serine proteases, and L-amino acid oxidases, which are known to contribute to skeletal muscle damage through membrane disruption, induction of oxidative stress, extracellular matrix degradation, and activation of inflammatory pathways.

To further assess venom potency against differentiated myotubes, both the maximum effect observed at the highest concentration tested (E_max_) and the concentration required to reduce cell viability by 50% (CC_50_) were determined (Table 1). Interestingly, *B. pauloensis* displayed the highest cytotoxic activity among all venoms evaluated, exhibiting a CC_50_ value below 5.00 µg mL^−1^ and the highest E_max_ value (91.0%), indicating a remarkable ability to compromise myotube viability. Similarly, *B. leucurus* showed substantial cytotoxicity, with a CC_50_ of 5.00 µg mL^−1^ and an E_max_ of 85.6%.

Given the pronounced cytotoxic effects of *B. pauloensis* venom (the only venom exhibiting both CC_50_ < 5 µg mL^−1^ and E_max_ > 90%) on differentiated myotubes, this venom was excluded from subsequent experiments evaluating cytotoxicity against murine macrophages and the different developmental forms of the parasites. This decision was made in accordance with the screening protocol previously adopted by our research group for the selection of promising compounds, with the aim of prioritizing venoms exhibiting a more favorable cytotoxicity profile, thereby enabling a more accurate assessment of their antiparasitic potential and selectivity.

### 2.3. Antiprotozoal Activity Against Promastigote Forms of L. amazonensis and L. infantum, and Epimastigote Forms of T. cruzi, as Well as Cytotoxicity Against Murine Peritoneal Macrophages

Initial screening against extracellular parasite forms is widely employed for the primary evaluation of antiprotozoal compounds, preceding the more labor-intensive and resource-demanding assays required to assess leishmanicidal activity against intracellular amastigote forms [30]. In this assay, except for *B. pauloensis*, all venoms were tested against the extracellular forms of the parasites, including promastigotes of *L. amazonensis* and *L. infantum*, as well as epimastigotes of *T. cruzi*.

As shown in Table 2, the most promising venoms were those from *B. alternatus* against *L. infantum*; *B. jararacussu* against both *L. infantum* and *L. amazonensis*; and the MTL and CTX pools from *C. durissus ruruima* against *L. amazonensis*. These venoms were therefore selected for further evaluation of cytotoxicity in murine peritoneal macrophages.

The selectivity index (SI) is an important parameter for identifying promising candidates for further evaluation of leishmanicidal activity against intracellular amastigotes, as murine peritoneal macrophages serve as the host cell model for infection. Thus, a suitable safety margin is essential when selecting compounds for further investigation, with priority generally given to candidates exhibiting SI values > 10 [31].

### 2.4. Antiamastigote Activity Against L. amazonensis

Based on the screening results presented in Table 2, two crude *C. durissus ruruima* venom samples exhibiting distinct compositional profiles (MTL and CTX) [32] exhibited the highest selectivity indices (SIs) against *L. amazonensis* promastigotes and were therefore selected for further evaluation against the intracellular amastigote stage. As shown in Table 3, both pools exhibited SI values > 10, meeting the predefined criterion for selection of candidates with a favorable selectivity profile.

Notably, the CTX pool displayed a higher SI than the MTL pool, with a value approaching that observed for the reference drug amphotericin B. Although the MTL pool showed comparatively lower selectivity, both pools exhibited low IC_50_ values against intracellular amastigotes, the clinically relevant stage of *Leishmania* responsible for infection of host cells. Collectively, these findings support the antileishmanial potential of *C. durissus ruruima* venom, and particularly its MTL- and CTX-enriched pools, warranting further investigation of their active components and underlying mechanisms of action.

## 3. Discussion

Among the sources explored for antiprotozoal drug discovery, snake venoms have emerged as particularly promising bioprospecting platforms, given the remarkable diversity and complexity of their molecular repertoire [12]. Systematic reviews and experimental studies have consistently documented significant antiprotozoal activity of crude venoms and their isolated fractions against multiple developmental forms of *Leishmania* species and *T. cruzi*, including promastigotes, amastigotes, epimastigotes, and trypomastigotes [33].

Among the major venom components with demonstrated antiprotozoal activity, phospholipases A2 (PLA2) stand out as one of the most extensively studied protein families. These enzymes exert their effects primarily through the hydrolysis of membrane phospholipids, generating lysophospholipids and free fatty acids that disrupt the structural integrity and selective permeability of parasite cell membranes, thereby compromising parasite viability and growth in a dose-dependent manner [34]. L-amino acid oxidases (LAAOs), in turn, are FAD-dependent flavoenzymes that catalyze the stereospecific oxidative deamination of L-amino acids to their corresponding α-keto acids, with the concomitant production of hydrogen peroxide (H_2_O_2_) and ammonia [35]. The H_2_O_2_ generated by this reaction is channeled to the surface of the enzyme via a structural hydrophobic tunnel and delivered to the immediate microenvironment of target cells, inducing localized oxidative stress that drives parasite cell death through apoptosis or necrosis, as demonstrated for *L. amazonensis* and *T. cruzi* [36]. Moreover, snake venom metalloproteases (SVMPs), zinc-dependent enzymes that represent major components of viperid and are also important for crotalid venoms, have also been explored for their antiprotozoal potential, with evidence suggesting that their proteolytic activity may disrupt surface proteins and extracellular matrix interactions critical for parasite infectivity and host cell invasion [37].

Widely present in *Bothrops* spp., snake venom serine proteases (SVSPs) constitute a superfamily of proteolytic enzymes capable of cleaving and inactivating biologically active proteins [38]. Regarding *Leishmania* spp., there is a scarcity of studies directly investigating the activity of SVSPs from *Bothrops* spp. and *Crotalus* spp. venoms. Other molecules that comprise *Bothrops* and *Crotalus* venoms, albeit in minor proportions, are the Cysteine-Rich Secretory Proteins (CRISPs), which can block ion channels and consequently alter cellular signaling [39,40]. Although few studies have addressed the antiparasitic activity of CRISPs, Adade et al. (2014) [41] demonstrated that the CRISP crovirin exhibits an IC_50_ value below 2 µg mL^−1^ against *L. amazonensis* and *T. cruzi* amastigotes. Another group of promising molecules with extensive pharmacological potential that has been gaining prominence in the therapeutic context consists of low-molecular-weight peptides. In the antiparasitic context, certain peptides from *B. atrox* and *C. d. terrificus* venoms are currently being investigated, such as batroxicidin and crotalicidin, which are both are cathelicidins (i.e., antimicrobial peptides) that exhibit promising activity against *L. amazonensis* and *T. cruzi* [42,43,44,45].

In this study, the venom protein profiles revealed a substantial pool of molecules in the crude venoms, consistent with the literature reports highlighting the presence of proteins predominantly within the 12–70 kDa and below 100 kDa ranges [7,46]. Notably, distinct differences were observed between the pools (MTL and CTX) of *C. d. ruruima*. Both CTX and MTL displayed a distinct band between approximately 52 and 76 kDa, which was more pronounced in the MTL pool. Thus, this different band is compatible with the molecular weight of class III snake venom metalloproteinases (PIII-SVMPs) [47]. The SVMP family comprises three main classes: PI, PII, and PIII. PI-SVMPs feature a single metalloproteinase domain; PII-SVMPs contain a metalloproteinase domain adjacent to a disintegrin domain; and PIII-SVMPs possess a catalytic metalloproteinase domain, a disintegrin-like domain, and an additional cysteine-rich domain, representing the most complex class among the three [37,48,49].

According to venomics approaches, PII- and PIII-SVMP classes have been described in the venom of *C. d. ruruima* [32,47,48]. In the metalloproteinase-enriched pool (MTL) of *C. d. ruruima*, we observed a signal between 76 and 102 kDa, along with a prominent signal between 52 and 76 kDa. These bands are consistent with the molecular weights of PIII-SVMP, reported in the literature under reducing conditions, suggesting an enrichment of these protein components [47]. However, other key molecules migrate within this same molecular mass range, such as serine proteinase Gyroxin-like (56 kDa) and L-amino acid oxidase (56 kDa) [47]. However, given the resolution limits of the gel, this association remains a tentative hypothesis pending mass spectrometry validation.

Beyond experimental limitations, factors such as age, sex, diet, habitat, and geography drive intraspecific variability and ontogenetic shifts in venom composition and activity. In the species studied here, these interconnected variables are well documented: habitat dictates prey availability, and diet shifts with body size across development [50,51]. For example, *B. leucurus* venoms vary in protein concentration across life stages, showing higher complexity in juveniles [52], while *B. alternatus* exhibits geographic variation in coagulant, proteolytic, and myotoxic potencies [53]. In *B. jararaca*, ontogenetic differences in lethality, sexual size dimorphism, female-biased venom yield and potency, and captive versus wild conditions are thoroughly described [54,55,56]. Similarly, adult *B. jararacussu* venoms display heightened myotoxicity [57], and *B. atrox* shows habitat- and age-dependent variation [58,59]. Parallel trends occur in *Crotalus*: *C. durissus terrificus* venom increases in protein concentration with age and varies regionally, a pattern mirrored in *C. d. ruruima* [60,61,62].

Given the confirmed presence of intact proteins and the lack of sample degradation, subsequent assays were performed against differentiated C2C12 myotubes, murine macrophages, *L. amazonensis* and *L. infantum* promastigotes, and *T. cruzi* epimastigotes as an initial screening. However, the literature reports have documented mild antiparasitic activity for BnSP-7—a myotoxic PLA2 from *B. pauloensis*—with an IC_50_ of 58.7 µg mL^−1^ against *L. amazonensis* [63]. Additionally, Bp-LAAO, an L-amino acid oxidase from the same species, has been tested against four different *Leishmania* species (*L. amazonensis*, *L. donovani*, *L. braziliensis*, and *Leishmania major*), yielding IC_50_ values consistently below 2 μg mL^−1^ (1.48, 1.59, 1.03 and 1.29 μg/mL, respectively) [64].

Regarding the cellular assays, the test conducted on differentiated myotubes demonstrated that the crude venom of *B. pauloensis* exhibits the highest toxicity among all crude venoms tested (CC_50_ = 3.9 μg mL^−1^; Figure 2F; Table 1). Alongside *B. pauloensis*, *B. leucurus* exhibited an CC_50_ value below 10 µg mL^−1^ (9.1 µg mL^−1^). Previous studies have also demonstrated the myotoxic activity of *B. leucurus* venom, characterized by pronounced local edema, histological evidence of muscle damage, and elevated serum CK levels. These effects are primarily driven by its PLA2 isoforms, such as Bleu-PLA2-like, Bleu TX-III, and blD-PLA2 [65,66,67,68]. Notably, the venoms that induced the highest reduction in differentiated C2C12 myotube viability are rich in PLA2s, with both *B. pauloensis* and *B. leucurus* interestingly sharing a venom composition highly abundant in these enzymes compared to other typical snake venom proteins such as SVMPs, LAAOs, C-type lectins, serine proteases, and low-molecular-weight peptides [68,69].

Conversely, the remaining *Bothrops* venoms tested do not feature PLA2s as their primary quantitative component, with the exception of *B. jararacussu*—which is also rich in these enzymes—with *B. atrox*, *B. jararaca*, and *B. alternatus* exhibiting SVMPs as their most abundant protein family [70,71,72,73]. Although the myotoxic activity of *B. jararacussu* was not as high as those of *B. pauloensis* and *B. leucurus*, it still ranked as the third most toxic venom tested (CC_50_ = 27.6 µg mL^−1^), converging with studies by Patrão-Neto et al. (2013) [74] and Saturnino-Oliveira et al. (2012) [75] that demonstrate myotoxicity through elevated serum CK levels and the edematogenic effect of *B. jararacussu* venom. Furthermore, the three other *Bothrops* venoms rich in SVMPs were also myotoxic against differentiated myotubes, presenting CC_50_ values ranging from 35 to 50 µg mL^−1^ (Table 1). This aligns with the literature reporting myotoxic activity characterized by local effects such as edema, serum CK elevation, and myonecrosis, alongside predominantly high hemorrhagic activity, resulting from vascular disruption caused by SVMPs [74,76,77,78,79].

Regarding snake venoms from the genus *Crotalus*, a plurality of results is observed concerning myotoxicity. *C. d. terrificus* venom is known to induce intense myotoxic damage, particularly in vivo, characterized by systemic skeletal muscle lesions accompanied by severe muscle pain and severe myocardial injury, along with elevated serum CK levels [80]. However, in vitro assays utilizing differentiated myotubes yielded a CC_50_ value of 148.6 µg mL^−1^, making it the venom with the lowest myotoxic activity among those tested. Consistently, an isolated PLA2 from *C. d. terrificus* tested in an in vitro C2C12 myoblast model failed to demonstrate an exacerbated reduction in cell viability and even induced cellular proliferation at low concentrations [81]. Furthermore, another study demonstrated that crotoxin isolated from *C. d. terrificus* was toxic toward C2C12 myoblasts, but not against differentiated C2C12 myotubes [82]. This same phenomenon is observed with the enriched pool of *C. d. ruruima* CTX, which exhibited the second lowest myotoxic activity against myotubes (CC_50_ = 91.8 µg mL^−1^). Despite being an enriched pool of crotoxin—a heterodimeric, notably myotoxic protein composed of two subunits, a basic PLA2 (CB) and an acidic crotapotin (CA)—its functionality in differentiated C2C12 myotubes did not reflect the muscle damage observed in vivo [8,83,84]. This discrepancy may be related to the inherent limitations of in vitro models, which do not fully replicate the physiological factors involved in crotoxin myotoxicity, such as the three-dimensional architecture of muscle tissue, interactions with extracellular matrix components, the presence of inflammatory mediators, and the complexity of neuromuscular interactions [85,86]. Additionally, although the CB subunit retains independent catalytic activity, the full expression of crotoxin toxicity appears to depend on the cooperative interaction between the CA and CB subunits, as well as specific physiological conditions that may be absent in the experimental system employed [84]. Although crotoxin is recognized as the primary myotoxic component of *C. d. ruruima* venoms, the enriched CTX pool did not induce the same degree of damage observed for the MTL pool in the C2C12 model (CC_50_ = 31.9 μg mL^−1^, respectively). Interestingly, however, the magnitude of the observed cytotoxicity was like those described for *B. atrox* and *B. alternatus* venoms (37.8 and 35.2 μg mL^−1^), in which SVMPs rank among the major venom constituents. Pharmacokinetic assessments, including absorption, distribution, metabolism, and excretion, as well as systemic toxicity assessments in animal models, should be addressed in future studies as subsequent steps in the preclinical evaluation of venom-derived molecules showing promising antiparasitic activity. In the present study, safety was initially assessed using two cellular models: murine peritoneal macrophages and C2C12 myotubes. However, further assays addressing neurotoxicity and hemotoxicity are warranted, as these represent important aspects of venom-induced pathology and are commonly evaluated in the characterization of snake venom effects and antivenom neutralization, including coagulation, hemorrhagic, hemolytic, and neuromuscular blockade activities [87,88,89]. Snake venoms of the genus *Bothrops*, for instance, are known to cause pronounced alterations in the hemostatic system, including procoagulant and anticoagulant activities, as well as proteolytic and hemorrhagic effects, to which SVMPs contribute substantially [70,71,72,73,89]. In contrast, *Crotalus* venoms are predominantly characterized by neurotoxic and myotoxic activities, although alterations in hemostasis may also occur, including effects on fibrinogen and coagulation pathways [90,91,92].

Regarding antiparasitic activity, both crude venoms from *B. atrox* and *C. d. terrificus* showed no activity against *L. amazonensis* or *L. infantum* promastigotes, nor against *T. cruzi* epimastigotes. However, it has been previously reported that an isolated and purified LAAO from *B. atrox* (BatroxLAAO) exhibited activity below 10 μg mL^−1^ against *L. donovani* and *L. major* promastigotes, with IC_50_ values of 4.3 μg mL^−1^ and 4.5 μg mL^−1^, respectively [93]. In *L. braziliensis* promastigotes, BatroxLAAO showed an IC_50_ above 10 μg mL^−1^ (IC_50_ = 23.34 μg mL^−1^), whereas *T. cruzi* trypomastigotes were less sensitive to it (IC_50_ = 62.8 μg mL^−1^) [93]. Batroxicidin, a cathelicidin-related antimicrobial peptide (AMP) isolated from *B. atrox* venom, displays antiparasitic effects with an IC_50_ of 0.44 μM against *T. cruzi* trypomastigotes and an EC_50_ of 4.90 μM against *L. amazonensis* promastigotes [45,47,94,95,96]. Crucially, crude venom contains a complex mixture of molecules that can mask the specific activities of individual toxins; therefore, biological activities observed for purified proteins are not always reproducible when the whole venom is evaluated [47,96]. Additionally, venom composition can vary based on biological and environmental factors, such as diet, age, habitat, and collection conditions, thereby influencing the relative abundance of specific components [97,98].

A similar pattern is observed for *C. d. terrificus*, as several of its isolated components and nanoformulations show reported antiparasitic activity [99,100,101,102,103,104,105]. Although isolated crotamine was inactive against intracellular *L. amazonensis* amastigotes, its incorporation into polylactic-glycolic acid (PLGA) particles [99] or combination with Glucantime^®^ [103] significantly reduced parasite numbers. Crotamine also acted synergistically when combined with amphotericin B against *L. amazonensis* promastigotes, unlike isolated crotamine [104]. Crotoxin isolated from *C. d. terrificus*, a molecule present and widely documented in venoms of the *Crotalus* genus, with multiple isoforms, was tested by Farias et al. (2017) [101] against *L. amazonensis* promastigotes and presented an IC_50_ of 22.86 μg mL^−1^ [106]. Likewise, Barros et al., 2015 [100] demonstrated that both *C. d. terrificus* PLA2s and a peptide fraction showed activity against *L. infantum* (IC_50_ = 52.07 and 16.98 μg mL^−1^, respectively). Because the literature on the crude venom is scarce, its lack of activity against *Leishmania* and *T. cruzi* in our study cannot be directly compared to isolated toxins; crude venom is a complex mixture where molecular interactions can modulate or mask specific biological effects.

The venoms that exhibited activity against all three parasites were the enriched pools of *C. d. ruruima*, as well as the crude venoms of *B. jararacussu* and *B. leucurus*. Among these, the most notable are those presenting IC_50_ values below 10 μg mL^−1^, such as *B. jararacussu* venom, which exhibits IC_50_ values of 2.9 and 2.8 μg mL^−1^ against *L. amazonensis* and *L. infantum* promastigotes, respectively, as well as significant anti-epimastigote activity against *T. cruzi* (IC_50_ = 15.5 μg mL^−1^; Table 2). Interestingly, the antiparasitic activity of an L-amino acid oxidase isolated from *B. jararacussu* venom, known as BjussuLAAO-II, has been previously reported against *L. amazonensis*, *L. braziliensis*, and *T. cruzi* [107,108]. Consistently, Barbosa et al. (2021) [108] reported compatible values, showing that, within the range of 1.56 to 12.5 μg mL^−1^, BjussuLAAO-II reduced the viable promastigote forms of *L. amazonensis* by 70% and *L. braziliensis* by 90%. Furthermore, a nanoformulation of an isolated *B. jararacussu* PLA2 (Asp49-PLA2) also showed in vitro activity against *L. amazonensis* promastigotes (IC_50_ = 14.36 μg mL^−1^) and intracellular amastigotes, and was able to reduce the in vivo parasite load by 73.5% in the lymph nodes of *L. amazonensis*-infected BALB/c mice [109,110]. The observed activity of the crude venom against *L. amazonensis* and *T. cruzi* may occur due to the presence of LAAOs and PLA2s, which represent 15% and 25.7% of the *B. jararacussu* venom composition, respectively, combined with the synergistic effects of other components, given that the crude venom consists of a complex mixture of toxins and bioactive molecules [73].

Both enriched pools from *C. d. ruruima* displayed strong anti-promastigote and anti-epimastigote activities. Notably, *C. d. ruruima* MTL exhibited great potency against *L. amazonensis*, *L. infantum*, and *T. cruzi*, with IC_50_ values of 1.6, 11.7 and 6.5 μg mL^−1^, respectively, surpassing Amphotericin B activity against *L. amazonensis* (IC_50_ = 3.3 μg mL^−1^). The *C. d. ruruima* CTX also showed great efficacy, especially on *L. amazonensis* promastigotes (IC_50_ = 3.8 μg mL^−1^). While the literature on the antiparasitic profile of *C. d. ruruima* venom is limited, the activity of isolated crotoxin from other *Crotalus* species against *L. amazonensis* promastigotes has been documented [101]. Although this toxin is a complex of two subunits with multiple isoforms that vary across species and subspecies, its inherent toxicity remains evident [8,84]. Data regarding the effects of isolated crotalic SVMPs on *Leishmania* spp. and *T. cruzi* remain limited, despite the promising antiparasitic activity observed here. While the exact mechanism of action was not explored in this study, it is possible that these proteins interfere with surface structures or essential processes for parasite survival. Thus, the presented data expand the knowledge on the biotechnological potential of *C. d. ruruima* venom and indicate SVMPs as promising candidates for future investigations.

While *B. leucurus* venom was not highly effective across all tested parasites, it showed reasonable activity against *L. amazonensis* promastigotes (IC_50_ = 15.4 μg mL^−1^). Studies investigating the antileishmanial or anti-chagasic properties of *B. leucurus* venom remain limited. Among the available literature, Torres et al. (2010) [111] isolated an L-amino acid oxidase from *B. leucurus* (BleuLAAO) and evaluated both the purified enzyme and the crude venom against *L. amazonensis* and *L. chagasi* promastigotes, as well as *T. cruzi* epimastigotes. Interestingly, BleuLAAO lacked inhibitory activity, but their crude venom exhibited high potency, with IC_50_ values of 1.94, 5.49 and 1.14 μg mL^−1^ for *L. amazonensis*, *L. chagasi*, and *T. cruzi*, respectively, suggesting that other venom components drive the observed effect [111]. Despite the differences in the IC_50_ values obtained in our study and by Torres et al. (2010) [111] against *L. amazonensis* (15.5 and 5.49 μg mL^−1^, respectively) and *T. cruzi* (49.4 and 1.14 μg mL^−1^, respectively), the results from both studies corroborate the antiparasitic potential of *B. leucurus* venom. The observed discrepancies may reflect the distinct methodologies employed to determine parasite viability; while our study utilized the colorimetric MTT assay to evaluate metabolic activity, Torres et al. (2010) [111] relied on direct cell counting in a Neubauer chamber. Additionally, Aranda-Souza et al. (2018) [112] demonstrated that a galactose-binding lectin (BLL) from *B. leucurus* exhibited micromolar activity against *L. amazonensis* and *L. braziliensis* promastigotes and amastigotes. Together, these findings highlight the pharmacological potential of *B. leucurus* venom, which clearly opens paths for further investigation.

Concurrently, some of the tested venoms demonstrated activity exclusively against *Leishmania* promastigotes, as observed for *B. alternatus* and *B. jararaca* crude venoms. Furthermore, except for the high potency of *B. alternatus* against *L. infantum* (IC_50_ = 7.7 μg mL^−1^), the activities of both *B. jararaca* and *B. alternatus* against *L. amazonensis* were lower than those of *B. jararacussu*, *C. d. ruruima* CTX, and *C. d. ruruima* MTL (Table 2). The literature regarding *B. jararaca* venom activity against *Leishmania* reveals divergent findings. Gonçalves et al. (2002) [29] reported IC_50_ values between 0.1 and 0.3 µg mL^−1^ against *L. major* promastigotes and *T. cruzi* epimastigotes over a 7-day monitoring period. In contrast, Deolindo et al. (2005) [113] observed a 50% growth inhibition of *T. cruzi* epimastigotes at 10 µg mL^−1^ on the fourth day of culture, indicating an activity about 30–100 times lower than that previously described [29,113]. Subsequently, Deolindo et al. (2010) [114] isolated eight distinct LAAOs (FI to FVIII) from *B. jararaca* venom, noting significant anti-epimastigote activity, particularly for the FI fraction, which reduced viability by 50% at 2.4 µg mL^−1^ and by 96% after 24 h. Nonetheless, these values remain lower than those reported by Gonçalves et al. (2002) [29,115], who notably employed fresh venom, potentially preserving bioactive components susceptible to degradation during processing and storage. Alternatively, Ciscotto et al. (2009) [115] evaluated the crude venom and an LAAO-rich fraction (HTP1) against *L. amazonensis* promastigotes, reporting viability reductions of only 31% and 52.5%, respectively, at a fixed concentration of 0.8 mg mL^−1^. These results are substantially lower than those observed in previous studies and in the present work (IC_50_ = 36.2 µg mL^−1^), highlighting considerable variability among studies. Together, the available data suggest that *B. jararaca* venom possesses genuine antiparasitic activity, although its magnitude is strongly influenced by experimental models, parasite species evaluated and venom preparation method.

For *B. alternatus*, the literature is even more scarce, not only for *Leishmania* and *Trypanosoma*, but also for protozoan parasites from other groups, such as *Plasmodium* spp. Although comparative studies are limited, the crude venom showed strong activity against *L. infantum* promastigotes, with IC_50_ values below 10 µg mL^−1^ and around 50 µg mL^−1^ for *L. amazonensis* promastigotes, with no activity against *T. cruzi*. Considering its venom composition, *B. alternatus* is characterized by a high abundance of SVMPs, reaching nearly 50% of its total composition, followed by serine proteases, PLA2s and LAAOs, as well as other bioactive molecules that have their antiparasitic effects already mentioned and may contribute, individually or synergistically, to the activity observed in the present study [71]. The initial screening of the crude venoms was performed using promastigote and epimastigote forms as a preliminary strategy to assess their antiparasitic activity. This approach allowed the identification of venoms with promising activity while limiting the number of samples subjected to more complex and resource-intensive assays. Thus, the use of these parasite forms in the initial screening contributed to the rational selection of samples for further investigation, while more clinically relevant parasite stages and appropriate biological models may be considered in future studies.

With this primary evaluation of the venoms, those that presented the best inhibitory activity (IC_50_ < 10 µg mL^−1^) were subjected to cytotoxicity evaluation in murine peritoneal macrophages, which are canonical cells for intracellular amastigotes. This assay is important to determine the selectivity index of the venoms, as promising antiparasitic activity must be accompanied by low toxicity to host cells, with selectivity being a fundamental parameter for prioritizing therapeutic candidates [31]. Of the four tested venoms (*B. alternatus*, *B. jararacussu*, *C. d. ruruima* MTL, and *C. d. ruruima* CTX), only *B. jararacussu* demonstrated a reduction in cell viability (CC_50_ = 197.5 µg mL^−1^) within the tested concentration range (4.7–300 µg mL^−1^), while the others maintained viability above 50% even at the highest concentration evaluated. It is known that *Bothrops* and *Crotalus* venoms play an important role in modulating the inflammatory response of macrophages, inducing the production of pro-inflammatory mediators [116,117,118,119,120,121]. Thus, despite the cytotoxic activity of *B. jararacussu*, the selectivity indices were significantly high—39 for *B. alternatus* against *L. infantum*; 68.1 and 70.5 for *B. jararacussu* against *L. amazonensis* and *L. infantum*, respectively; and 187.5 and 78.5 for *L. amazonensis* against *C. d. ruruima* MTL and *C. d. ruruima* CTX, respectively—even more so when compared to Amphotericin B (SI = 30.2 against *L. amazonensis*), which is one of the standard drugs for leishmaniasis treatment.

Thus, the two highest SIs were selected for the evaluation of anti-amastigote activity, which is the form found in the vertebrate host and clinically important in the therapeutic context [122]. Therefore, the venoms of *C. d. ruruima* MTL (SI = 187.5) and *C. d. ruruima* CTX (SI = 78.9) were subjected to the assay against intracellular amastigotes of *L. amazonensis* within the concentration range of 0.3 to 10 µg mL^−1^. Both presented activity against this parasitic form, indicating that the effect previously observed in promastigotes also extends to the clinically relevant form of the parasite. Although the CTX pool presented a greater inhibitory effect in numerical terms (IC_50_ = 2.5 µg mL^−1^), no statistically significant differences were observed compared to the MTL pool (IC_50_ = 4.1 µg mL^−1^; unpaired t test, *p* > 0.05). In this way, the results suggest that both components enriched in crotoxin and those enriched in metalloproteinases can contribute to the observed anti-amastigote activity. These findings provide preliminary evidence supporting the potential of *Crotalus* venoms as a source of antiparasitic molecules and warrant further investigation, particularly through fractionation and molecular characterization studies, to identify the main compounds responsible for this effect and to determine whether isolated molecules exhibit greater potency and selectivity than the evaluated venom pools. This characterization is important for guiding subsequent in vivo studies to further support the observed anti-amastigote activity, assess parasite burden reduction, evaluate the normalization of biochemical parameters, and determine whether venom-derived components have therapeutic potential.

## 4. Conclusions

In conclusion, this study reinforces Brazilian toxinological biodiversity as a valuable source for the discovery and development of novel therapeutic agents against neglected tropical diseases (NTDs). Our findings demonstrate that, although crude venoms from *Bothrops* species exhibited pronounced cytotoxicity toward mammalian cells, *C. durissus* was able to dissociate cellular toxicity from potent antiparasitic activity. Notably, *C. durissus ruruima* emerged as the most promising subspecies, with its MTL (metalloprotease-rich) and CTX (crotoxin-rich) pools displaying exceptional selectivity indices (SI = 187.5 and 79.9, respectively, against *L. amazonensis* promastigotes) and an unprecedented ability to eliminate intracellular amastigotes at low IC_50_ values (4.1 and 2.5 µg mL−1 for MTL and CTX, respectively). Furthermore, both exhibited high intracellular selectivity, with intracellular selectivity indices (SIs) of 73 and 120 for MTL and CTX, respectively. Our findings not only validate the biotechnological potential of snake venoms from the Brazilian fauna, but also provide a robust foundation for future investigations aimed at isolating and characterizing their bioactive components and further preclinical studies to assess efficacy and safety in appropriate animal models. Such efforts may ultimately enable the identification of novel molecular scaffolds for the rational design of next-generation antiprotozoal agents with improved safety, selectivity, and therapeutic efficacy.

## 5. Materials and Methods

### 5.1. Venoms

The venoms of *B. jararacussu*, *B. pauloensis*, *B. alternatus*, *B. leucurus*, *B. atrox*, *B. jararaca*, and *C. durissus terrificus* were provided by the Center for the Study of Venoms and Venomous Animals of UNESP (CEVAP, Botucatu, SP, Brazil), under CRMV Registration No. 47038 and ART No. 05940/2024. The facility is registered with the Institutional Animal Care and Use Committee (CEUAIBTEC/UNESP; CNPJ 48.031.918/0001-24) under License CIAEP No. 02.0472.2022 (issued on 24 June 2022), with authorization under Process No. SMA 000000002175/2013 and Wildlife Management Authorization No. 0000118253/2024. The specimens were collected from various municipalities in the states of São Paulo, Minas Gerais, and the Federal District, encompassing a broad geographical distribution. The samples were subsequently analyzed at the Center for the Study of Venoms and Venomous Animals (CEVAP) of São Paulo State University (UNESP), situated in Botucatu, São Paulo, Brazil. The metalloprotease-enriched (MTL) and crotoxin-enriched (CTX) pools from *C. durissus ruruima* venom were obtained in Boa Vista, Roraima, Brazil, by the Snakebite Roraima research group, under ICMBio/SISBIO permit No. 79102-4, and were previously investigated and chemically characterized in a published study (more detail about the enriched pool can be obtained elsewhere) [32,91]. All venom samples were stored at −20 °C, protected from light, until use.

### 5.2. Electrophoresis

The venom composition was analyzed by polyacrylamide gel electrophoresis (Sodium Dodecyl Sulfate–Polyacrylamide Gel Electrophoresis, SDS-PAGE), under denaturing and reducing conditions. Protein separation was performed using a 12% resolving gel and a 5% stacking gel. Venom samples were prepared at a 1:1 ratio with sample buffer (50 mM Tris-HCl, pH 6.8, 25% SDS, 10% glycerol, 0.002% bromophenol blue). A total of 20 µg of venom protein per lane was loaded onto the gel. Electrophoresis was performed at a constant voltage of 120 V until adequate protein separation was achieved. Gels were stained with Coomassie Brilliant Blue R-350 (PlusOne Coomassie Blue PhastGel^®^, GE Healthcare, Chicago, IL, USA) [32].

### 5.3. Parasite Culture

*L. amazonensis* (strain MPRO/BR/1972/M1841-LV79) promastigotes and *T. cruzi* (cepa Y) epimastigotes were cultured in Liver Infusion Tryptose (LIT) medium (pH 7.5) supplemented with 13.4 µg mL^−1^ penicillin, 0.2 mg mL^−1^ streptomycin (Sigma), and 10% (*v*/*v*) heat-inactivated fetal bovine serum (FBS; Thermo, Grand Island, NY, USA) at 26 °C [123,124].

*L. infantum* (strain MHOM/MA/67/ITMAP-263) promastigotes were cultured in 199 (EARLE) medium (pH 7.5) supplemented with 13.4 µg mL^−1^ penicillin, 0.2 mg mL^−1^ streptomycin (Sigma), 0.01 M sodium bicarbonate (Sigma) solution, 1 M HEPES (Sigma) buffer solution, 10% (*v*/*v*) heat-inactivated fetal bovine serum (FBS) and 10% (*v*/*v*) male human urine at 26 °C [125].

### 5.4. Culture of C2C12 Cells and Induction of Differentiation into Myotubes

Murine C2C12 myoblasts (ATCC^®^ CRL-1772™), a subclone originally derived from the mouse myoblast cell line established by Yaffe and Saxel, were thawed from cryovials previously preserved in RPMI-1640 freezing medium supplemented with 10% DMSO. Cells were cultured in tissue culture flasks containing RPMI-1640 medium modified with L-glutamine (2.05 mM), penicillin–streptomycin (1%), β-mercaptoethanol (50 µM), HEPES (25 mM), and sodium bicarbonate (2.0 g/L), all obtained from Nova Biotecnologia, and supplemented with 10% heat-inactivated sterile fetal bovine serum (Bio Nutrientes, Taciba, São Paulo, Brazil). Cultures were maintained at 37 °C in a humidified atmosphere containing 5% CO_2_ until approximately 80% confluence was reached. Cell dissociation was performed using 0.25% trypsin containing 0.2 g/L EDTA diluted in 1× HBSS (Nova Biotecnologia, Cotia, São Paulo, Brazil). Following dissociation, cells were seeded into 96-well plates at a density of 0.5 × 10^5^ cells/mL in complete medium. On the first day after seeding, the supplementation was replaced with 2% horse fetal serum (Bio Nutrientes), and this condition was maintained until the third day with daily medium replacement. On the fourth day, when cells exhibited morphology consistent with myotube formation and reached approximately 70–80% confluence, treatments with the toxins were initiated [126].

### 5.5. Cytotoxicity Against Differentiated C2C12 Myotubes

To evaluate cytotoxicity against differentiated C2C12 myotubes, cells were exposed to different concentrations of the selected venoms for 24 h at 37 °C in an atmosphere containing 5% CO_2_. The venoms from *B. jararaca*, *B. jararacussu*, *B. alternatus*, *B. leucurus*, *B. pauloensis*, *B. atrox*, *C. durissus terrificus*, and the venom of *C. durissus ruruima* (MTL or CTX pools) were evaluated in different concentrations (120 µg mL^−1^ to 5 µg mL^−1^). After incubation, cell viability was determined using the colorimetric MTT assay (3-(4,5-dimethylthiazol-2-yl)-2,5-diphenyl-2H-tetrazolium bromide), based on the reduction of MTT to formazan crystals. No positive controls were used in this assay. The MTT working solution was prepared from a 5 mg mL^−1^ stock solution in PBS and subsequently diluted 1:9 in phenol red-free and serum-free RPMI-1640 medium, yielding a final concentration of 0.5 mg mL^−1^. The MTT solution was added to the cultures at a volume corresponding to 10% of the total well volume, followed by incubation for 3 h at 37 °C. Subsequently, the medium was removed, and the formazan crystals were solubilized with 100 µL of DMSO per well. Absorbance was measured at 570 nm using a microplate spectrophotometer. All experiments were performed in technical and biological octuplicates. Absorbance values were normalized relative to the negative control group, consisting of RPMI-1640 medium supplemented with 2% horse serum in the absence of venom treatment [127]. The obtained data were analyzed using GraphPad Prism 8.0.0 (GraphPad Software, LLC, Boston, MA, USA) software to determine the concentration required to reduce the viability of differentiated C2C12 myotubes by 50% (CC_50_), expressed in μg mL^−1^, as well as the maximum effect (E_max_), expressed as a percentage (%).

### 5.6. Antiprotozoal Activity Against Promastigote Forms of L. amazonensis and L. infantum, and Epimastigote Forms of T. cruzi

For determining the anti-trypanosomatid activity employed by MTT colorimetric assay, the MTT assay was based on the determination of the ability of living cells to reduce MTT to crystals of formazan. In this work, *L. amazonensis* and *L. infantum* promastigote, as well as *T. cruzi* epimastigote forms (1 × 10^7^ parasites/mL), were treated with the respective venoms and positive controls (benznidazole for epimastigotes and amphotericin B for promastigotes) at different concentrations to determine the half-maximal inhibitory concentration (IC_50_), expressed as µg mL^−1^. The compounds were tested against the extracellular parasites in different concentrations (100 µg mL^−1^ to 1.56 µg mL^−1^) and incubated at 27 °C for 72 h [30]. Following the incubation period, the formazan crystals were solubilized, and absorbance was measured in 96-well plates at 570 nm for *Leishmania* species and 595 nm for *T. cruzi* using an Infinite 200 PRO microplate reader (Tecan, Männedorf, Switzerland). Each concentration was tested in technical triplicate across three independent biological replicates. The safety index (SI) was calculated, and SI around 10 means that a compound can be better evaluated for further studies [31,128,129]. The data obtained were processed with the software GraphPad Prism 8.0.0 to calculate the IC_50,_ expressed in μg mL^−1^. It is important to note that, because the MTT assay measures metabolic activity, the data obtained in the present study do not allow discrimination between trypanosomatidicidal and trypanosomatistatic effects.

### 5.7. Isolation of Murine Peritoneal Macrophages

For the isolation of murine peritoneal macrophages, 1 mL of 3% sodium thioglycolate solution (Difco, Sparks, MD, USA) was injected into the peritoneal cavity of male Swiss mice aged 4–8 weeks to stimulate macrophage recruitment to the peritoneal region. Four days after stimulation, the animals were euthanized in accordance with procedures approved by the local Ethics Committee (approval no. 03/2019). Following euthanasia and abdominal asepsis with 70% ethanol, the peritoneal exudate was collected. Subsequently, cells were counted, 1 × 10^6^ murine macrophages were resuspended in complete Roswell Park Memorial Institute (RPMI-1640, Cultilab, Campinas, SP, Brazil) medium and seeded into sterile 96-well plates (for cytotoxicity assays), and 5 × 10^5^ macrophages were pipetted onto circular coverslips in 24-well plates (for anti-amastigote assays) and allowed to adhere for 4 h at 37 °C in a 5% CO_2_ atmosphere [30].

### 5.8. Cytotoxicity Against Murine Peritoneal Macrophage

Venoms that exhibited activity against the extracellular forms of the parasites were evaluated for cytotoxicity toward murine peritoneal macrophages (1 × 10^6^ cells/mL). *C. durissus ruruima* MTL and CTX, *B. jararacussu*, *B. pauloensis*, and *B. alternatus* venoms, as well as the positive controls (benznidazole and amphotericin B), were incubated with murine peritoneal macrophages at different concentrations (300 µg mL^−1^ to 4.68 µg mL^−1^) for 24 h in a 5% CO_2_ atmosphere and 37 °C. Following the incubation period, the formazan crystals were solubilized, and absorbance was measured in 96-well plates at 570 nm using an Infinite 200 PRO microplate reader (Tecan, Männedorf, Switzerland). Each concentration was tested in technical triplicate across three independent biological replicates. Absorbance values were normalized relative to the negative control group, consisting of RPMI-1640 medium supplemented with 10% FBS in the absence of venom treatment. The data obtained were processed with the software GraphPad Prism 8.0.0 to calculate the CC_50,_ expressed in μg mL^−1^. The selectivity index (SI) was calculated, and the highest SI values were observed for *L. amazonensis*, which was therefore prioritized for the anti-amastigote activity assay [123].

### 5.9. Antiamastigote Activity Against L. amazonensis

To determine anti-amastigote activity against *L. amazonensis*, adherent murine peritoneal macrophages, as described in Section 5.5, were incubated with stationary-phase promastigotes (parasite-to-macrophage ratio of 5:1 in RPMI medium; 2.5 × 10^6^ parasites/mL), in which highly infective metacyclic forms predominate, for up to 18 h at 37 °C in a 5% CO_2_ atmosphere to allow infection and differentiation into amastigote forms [111]. Following incubation, non-internalized parasites were removed by washing with PBS. The infected macrophages were then treated with varying concentrations (10.0 µg mL^−1^ to 0.312 µg mL^−1^) of venoms from *C. durissus ruruima* (MTL and CTX) in RPMI-1640 medium for 24 h at 37 °C under a 5% CO_2_ atmosphere. After that treatment, macrophages were washed with PBS, and coverslips were fixed with methanol at 5 min and cored with Giemsa 5% (*v*/*v*) at 8 min, after which we realized the counting of intracellular amastigotes per infected macrophage. Each concentration was tested in technical triplicate across three independent biological replicates. The data obtained were processed with the software GraphPad Prism 8.0.0 to calculate the IC_50,_ expressed in μg mL^−1^, and the selectivity index (SI) and Infection Index were calculated [31,48].

### 5.10. Statistical Analysis

In this study, an unpaired *t*-test was used for the statistical analyses of the cytotoxicity and antiparasitic activity assays. Statistical analyses were performed using GraphPad Prism version 8.0.0 (GraphPad Software, LLC), and a *p*-value < 0.05 was considered statistically significant.

## Figures and Tables

**Table 1:** Cytotoxic effects of snake venoms on differentiated C2C12 myotubes.

Treatment	CC_50_ * (µg mL^−1^)	E_max_ ** (%)
*B. alternatus*	35.2 ± 2.4	80.5 ± 4.2
*B. atrox*	37.8 ± 0.4	58.0 ± 0.5
*B. jararaca*	44.7 ± 4.3	72.3 ± 5.6
*B. jararacussu*	27.6 ± 3.1	76.8 ± 1.1
*B. leucurus*	9.1 ± 1.1	85.6 ± 0.6
*B. pauloensis*	3.9 ± 1.0	91.0 ± 0.2
*C. durissus terrificus*	148.6 ± 12.9	28.5 ± 6.0 ^#^
*C. durissus ruruima* MTL	31.9 ± 4.1	88.6 ± 0.3
*C. durissus ruruima* CTX	91.8 ± 14.5	54.6 ± 2.3

* 50% Cytotoxicity Concentration (CC_50_, µg mL^−1^) and ** maximum effect (E_max_, %) against differentiated C2C12 myotubes. CC_50_ values represent the concentrations estimated from the fitted concentration–response curves to produce a 50% reduction in cell viability. ^#^ For *C. durissus terrificus*, the CC_50_ value (148.6 ± 12.9 µg mL^−1^) was estimated by extrapolation of the fitted concentration–response curve, as the maximum observed effect (E_max_ = 28.5 ± 6.0%) did not reach 50% cytotoxicity within the tested concentration range.

**Table 2:** Antiprotozoal activities (IC_50_, µg mL^−1^) of the venoms against the extracellular forms of parasites, together with cytotoxicity (CC_50_, µg mL^−1^) toward murine macrophages, and corresponding selectivity indices (SIs).

Treatment	Extracellular Parasite Form			Murine Peritoneal Macrophages
	*L. amazonensis* Promastigote	*L. infantum* Promastigote	*T. cruzi* Epimastigote	
*B. alternatus*	48.4 ± 1.4 (6.2)	7.7 ± 0.4 (39)	>100	>300
*B. atrox*	>100	>100	>100	NA
*B. jararaca*	36.2 ± 1.6	>100	>100	NA
*B. jararacussu*	2.9 ± 0.3 (68.1)	2.8 ± 0.01 (70.5)	15.5 ± 1.3 (12.7)	197.5 ± 1.9
*B. leucurus*	15.4 ± 0.3	20.1 ± 1.5	49.4 ± 0.2	NA
*C. d. terrificus*	>100	>100	>100	NA
*C. d. ruruima* MTL	1.6 ± 0.3 (187.5)	11.7 ± 0.2 (25.7)	6.5 ± 0.2 (46.2)	>300
*C. d. ruruima* CTX	3.8 ± 0.3 (78.9)	12.4 ± 0.1 (24.1)	13.5 ± 0.2 (22.2)	>300
Amphotericin B	3.3 ± 0.2 (30.2)	5.4 ± 1.4 (18.4)	NA	99.8 ± 3.7
Benznidazole	NA	NA	4.1 ± 0.3 (241.0)	988.4 ± 38.1

NA, not assessed.

**Table 3:** Antiprotozoal activity (IC_50_, µg mL^−1^) of the *C. d. ruruima* venoms against the amastigote forms of the *L. amazonensis* and corresponding selectivity index (SI).

Treatment	*L. amazonensis* Amastigotes *
*C. durissus ruruima* (MTL)	4.1 ± 0.3 (73.1)
*C. durissus ruruima* (CTX)	2.5 ± 0.7 (120.0)
*Amphotericin B*	0.75 ± 0.1 (133.0)

* An unpaired *t*-test showed no statistically significant difference between the IC_50_ values for MTL and CTX (*p* > 0.05).

## Data Availability

The original contributions presented in this study are included in the article. Further inquiries can be directed to the corresponding authors.
